# Serological evaluation of risk factors for exposure to malaria in a pre-elimination setting in Malaysian Borneo

**DOI:** 10.1038/s41598-023-39670-w

**Published:** 2023-08-10

**Authors:** Isabel Byrne, Timothy William, Tock H. Chua, Catriona Patterson, Tom Hall, Mark Tan, Chetan Chitnis, John Adams, Susheel K. Singh, Lynn Grignard, Kevin K. A. Tetteh, Kimberly M. Fornace, Chris J. Drakeley

**Affiliations:** 1 Faculty of Infectious and Tropical Diseases, London School of Hygiene and Tropical Medicine, Keppel Street, Bloomsbury, London, WCIE 7HT UK; 2 Infectious Diseases Society Sabah-Menzies School of Health Research Clinical Research Unit, Kota Kinabalu, Malaysia; 3 Gleneagles Hospital, Kota Kinabalu, Malaysia; 4 Clinical Research Centre, Queen Elizabeth Hospital, Kota Kinabalu, Malaysia; 5 Faculty of Medicine and Health Sciences, Universiti Malaysia Sabah, Kota Kinabalu, Malaysia; 6 Department of Parasites and Insect Vectors, Malaria Parasite Biology and Vaccines, Institut Pasteur, Paris, France; 7 Center for Global Health and Infectious Diseases Research, College of Public Health, University of South Florida, Tampa, FL USA; 8 Centre for Medical Parasitology at Department of International Health, Immunology and Microbiology, University of Copenhagen, Copenhagen, Denmark; 9 Department of Infectious Diseases, Copenhagen University Hospital, Rigshospitalet, Copenhagen, Denmark; 10 School of Biodiversity, One Health and Veterinary Medicine, University of Glasgow, Glasgow, Scotland; 11 Saw Swee Hock School of Public Health, National University of Singapore, Singapore, Singapore

**Keywords:** Malaria, Risk factors

## Abstract

Malaysia has reported no indigenous cases of *P. falciparum* and *P. vivax* for over 3 years. When transmission reaches such low levels, it is important to understand the individuals and locations where exposure risks are high, as they may be at greater risk in the case of a resurgence of transmission. Serology is a useful tool in low transmission settings, providing insight into exposure over longer durations than PCR or RDT. We ran blood samples from a 2015 population-based survey in northern Sabah, Malaysian Borneo on a multiplex bead assay. Using supervised machine learning methods, we characterised recent and historic exposure to *Plasmodium falciparum* and *P. vivax* and found recent exposure to *P. falciparum* to be very low, with exposure to both species increasing with age. We performed a risk-factor assessment on environmental, behavioural, demographic and household factors, and identified forest activity and longer travel times to healthcare as common risk-factors for exposure to *P. falciparum* and *P. vivax.* In addition, we used remote-sensing derived data and geostatistical models to assess environmental and spatial associations with exposure. We created predictive maps of exposure to recent *P. falciparum* in the study area and showed 3 clear foci of exposure. This study provides useful insight into the environmental, spatial and demographic risk factors for *P. falciparum* and *P. vivax* at a period of low transmission in Malaysian Borneo. The findings would be valuable in the case of resurgence of human malarias in the region.

## Introduction

Malaysia has made strong progress in the prevention and control of malaria since the introduction of the Malaria Eradication Program in the 1960s. In 2011, the country introduced the National Malaria Elimination Strategic Plan and set the target of reaching the “malaria-free” status by 2020, here meaning the elimination of human indigenous malaria transmission. Malaysia was on track to achieve this goal for human malaria species *P. falciparum, P. malariae, P. ovale* and *P. vivax*^1^*.* There have been zero indigenous human malaria cases reported for over three years and zero malaria deaths since 2017^2^. In the past two decades, however, the emergence and increase in human cases of the zoonotic *P. knowlesi* has threatened the certification of Malaysia as malaria-free^2^. As the epidemiology of zoonotic malaria changes in Malaysia, it is important to track decreases and understand risks for human malarias alongside.

In the case of nonzoonotic, human malarias, as a country transitions from endemic to low-transmission and finally to an elimination setting, the epidemiology changes; infections become more spatially heterogenous and may be more difficult to detect and to diagnose. At this stage, classic passive surveillance systems which do not detect asymptomatic cases^3–5^, and cases in hard-to-reach communities or in populations who are unlikely or unable to visit health facilities^6,7^ may need supplementing with active surveillance. Active surveillance seeks out infections and asymptomatic parasite carriers, and can help to identify populations at higher risk of transmission^8^. Common active surveillance methods in malaria are cross-sectional sampling of communities using RDT diagnostics and PCR to detect active infections. Although they often identify asymptomatic cases, these are active infections and surveillance in low-transmission settings can benefit from approaches that improve burden estimates and understand risk-factors for exposure^7^.

Serological assays are one-such approach. They provide measurements of an individual’s antibody responses to a specific pathogen, which is used as a proxy for previous exposure. With a growing understanding of the specificity, longevity and duration of specific malaria biomarkers in the immune system, it is possible to characterise an individual’s recent and past exposure history to the human malarias *P. falciparum* and *P. vivax*^9–13^*.* Utilising multiplex bead assays (MBA) makes the measurement of serological responses at a population-level operationally feasible, as they only require small amounts of serum or plasma collected by finger-prick sampling in EDTA microcontainers to measure a broad range of immune responses^14,15^. Incorporating participant questionnaires in active surveillance surveys with serology can provide information on the anthropological factors impacting risk of exposure in populations. Understanding the factors underpinning exposure in higher risk populations may provide guidance for policy in the remainder of the country, which may be useful in the mitigation of human malaria resurgence in the future^4,8^.

Geostatistical methods can be applied to serological response data to understand the spatial heterogeneity of malaria exposure. By relating infection metrics with environmental and spatial covariates, geostatistical mapping can predict disease burden in unsampled space, identifying areas of higher exposure risk and receptive areas at risk of outbreaks^7,14,16,17^. Geostatistical methods are increasingly being applied in malaria research to identify environmental risk-factors for exposure and map incidence, prevalence, or other burden metrics^18–20^. This is especially useful in areas of low-transmission where infection data is sparse and transmission is spatially heterogenous. The use of serology data in geostatistical mapping exercises has previously come with challenges, where serological markers which represent historical exposure are linked to data representing current environmental and spatial conditions^14^. Additionally, when mapping *P. vivax* exposure the potential for relapses due to the reactivation of hypnozoite stage parasites should be considered. Differentiation between novel and relapse cases in *P. vivax* remains a challenge in mapping, as antibody production may be occurring in a different location to the original site of exposure^18,21^. Mapping historic exposure or *P. vivax* exposure can therefore result in temporally inconsistent patterns of exposure and risk being mapped. For this reason, geostatistical methods are currently better suited to mapping novel recent or current exposure to malaria antigens. These methods can be used to produce maps of uncertainty in predictions, which can highlight areas to prioritise for data collection^19^.

To examine the risk of exposure to nonzoonotic malaria, we analysed blood-samples collected during a 2015 ecologically stratified cross-sectional survey in Northern Sabah, Malaysia using a serology MBA. The survey was conducted closely prior to the last reported indigenous cases of *P. falciparum* and *P. vivax.* We aimed to estimate short and long-term population exposure to *P. falciparum* and *P. vivax* based on antibody profiles, and to assess the individual and household-level risk-factors for exposure to these species*.* As historical exposure may reflect past environmental conditions or residence locations, geostatistical methods are best fit to predict recent or novel current infection, therefore we only performed spatial analysis on predictions for recent exposure to *P. falciparum.* We aimed to find environmental and spatial risk-factors for exposure and used geostatistical methods to predict population-level exposure probabilities.

## Results

### Sample collection

**Figure 1:**
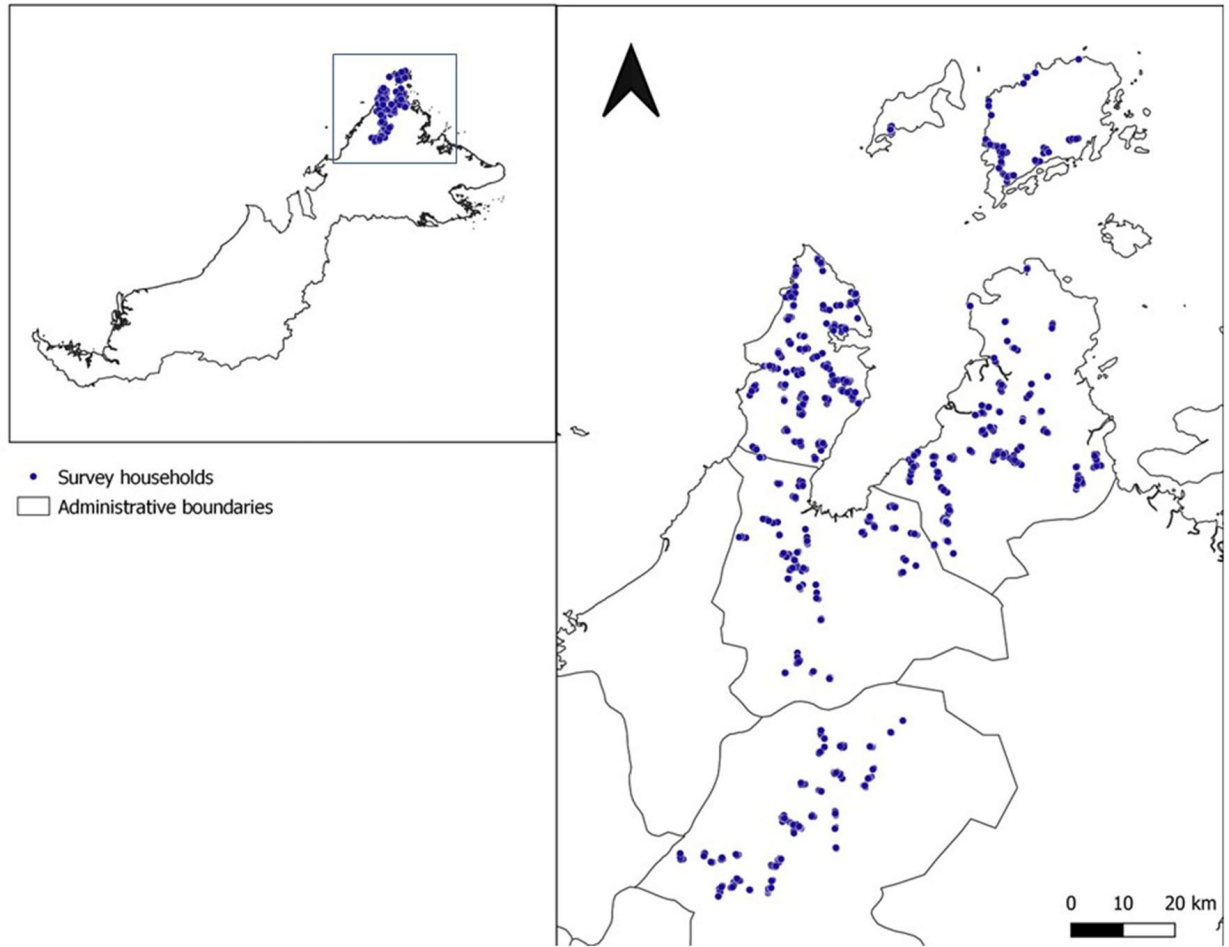
Right: Map of Malaysian Borneo. Left: Map of northern Sabah, Malaysian Borneo with survey households. Maps were created using QGIS (version 3.16.11)^24^.

10,100 individuals were sampled from 2849 households in 180 villages (Fig. 1), with ages ranging from 3 to 105 years old. The demographic breakdown of the sampled population and their malaria prevention measures are reported in Fornace et al*.*^22^.

### Diagnostics

**Figure 2:**
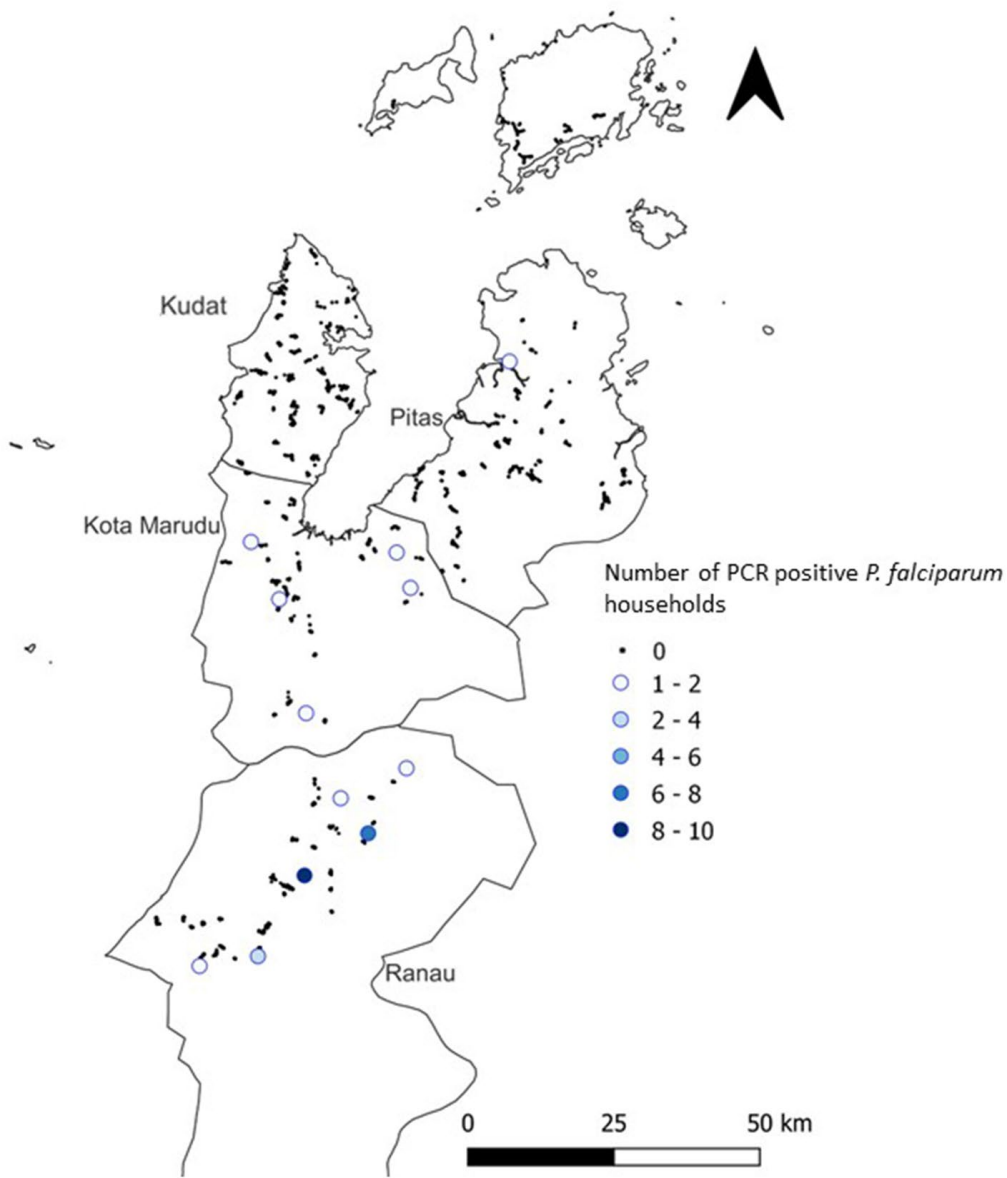
Map of P. falciparum PCR results. Maps were created using QGIS (version 3.16.11)^24^.

Despite 303 (3%) sampled individuals reporting fever, no individuals were identified as positive for malaria by microscopy. 55 (0.005%) individuals were positive for infection with *P. falciparum* by pooled PCR. Mono-infections were found for 3 individuals with *P. knowlesi,* three with *P. malariae,* and one with *P. vivax*^22^. Two mono-infections were also identified with the zoonotic malaria *P. cynomolgi*^23^*.* Additionally, one mixed-infection with *P. knowlesi* and *P. vivax* and one mixed-infection with *P. vivax* and *P. malariae* were found. One infection was identified with a *Plasmodium* species which could not be confirmed^22^. The mono-infections identified are listed in Table 1, and Fig. 2 presents a map of PCR results for *P. falciparum*. 3 village-level clusters of positive households were found. Cross-reactivity between these infections and antibody responses were not investigated as it was not possible to rule out previous exposure to other malaria species, all of which were circulating in the area at the time of the survey.

**Table 1:** Positive results for mono-infections from microscopy and PCR investigations.

Individuals sampled	Microscopy positive	PCR *P. falciparum* positive	PCR *P. vivax* positive	PCR *P. knowlesi* positive	PCR *P. malariae* positive	PCR *P. cynomolgi* positive
10,100	0	55	1	3	3	3

MFI data for 10,542 samples of known *P. falciparum* exposure status were used as training and validation data in the Super Learner models for classification of recent exposure to *P. falciparum* (Supplementary Table 1)*.*

The final combination of antigens used in the classification model were *P. falciparum* AMA1, MSP1-19, GLURP-R2, Etramp5, MSP2-Ch150/9, GEXP18 and SEA (Supplementary Fig. 1). The results of the tests for relative influence of each antigen are presented in Supplementary Table 2. The final SuperLearner weighted ensemble performed better than all individual algorithms except for the boosted regression tree (BRT) (Supplementary Information Table 3). While the BRT yielded predictions which were marginally more accurate than the final SuperLearner ensemble, > 500 predictions were classified as missing values. To maximize the classified data which we could use, we used predictions from the SuperLearner ensemble. The AUC values of each algorithm’s predictions are presented in the Supplementary Information Table 3. The final SuperLearner weighted ensemble model for to *P. falciparum* identified recently exposed individuals highly accurately with a cross-validated AUC of 0.996 (0.974–1). 406 individuals (0.04% prevalence, 95% CI 0.036–0.044) were predicted to be positive for recent exposure to *P. falciparum.*

**Figure 3:**
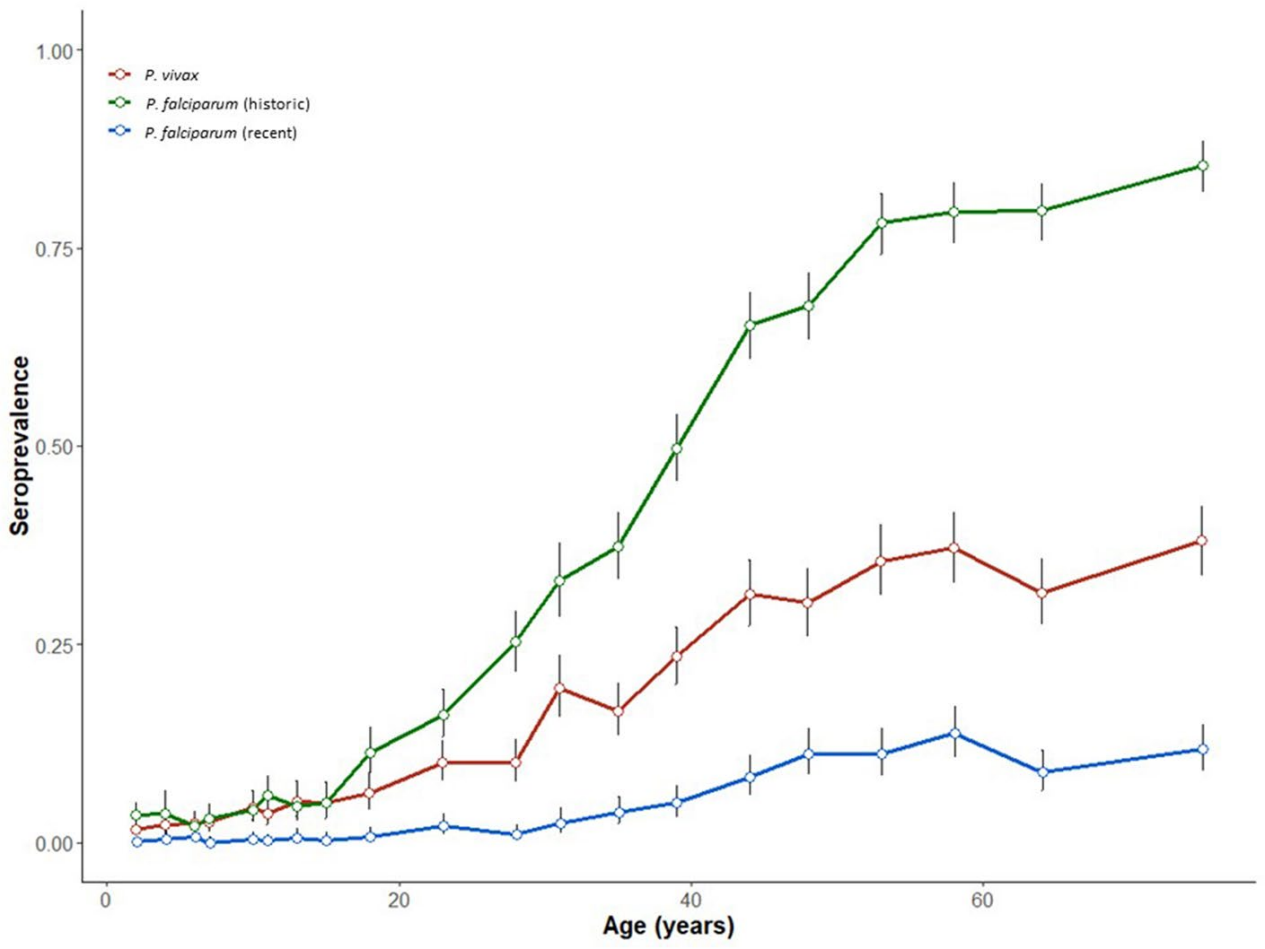
Age-stratified seroprevalence for exposure to *P. vivax* (red line), historic *P. falciparum* (green line), recent *P. falciparum* (blue line). Vertical lines represent confidence intervals for seroprevalence estimates.

Fornace *et al*^22^ used the SuperLearner methodology on the same test data to estimate historic exposure to *P. falciparum* and exposure to *P. vivax*. They used confirmed positives samples and adults from previously hyper-endemic areas as training data. Estimations from Fornace et al.^22^ found that historical exposure prevalence to *P. falciparum* was 32.4% (95% CI 31.4–33.4) and 16.4% (95% CI 15.6–17.1) for *P. vivax*. Exposure was positively associated with increasing age for both species (Fig. 3).

### Household-level risk factors for exposure

**Figure 4:**
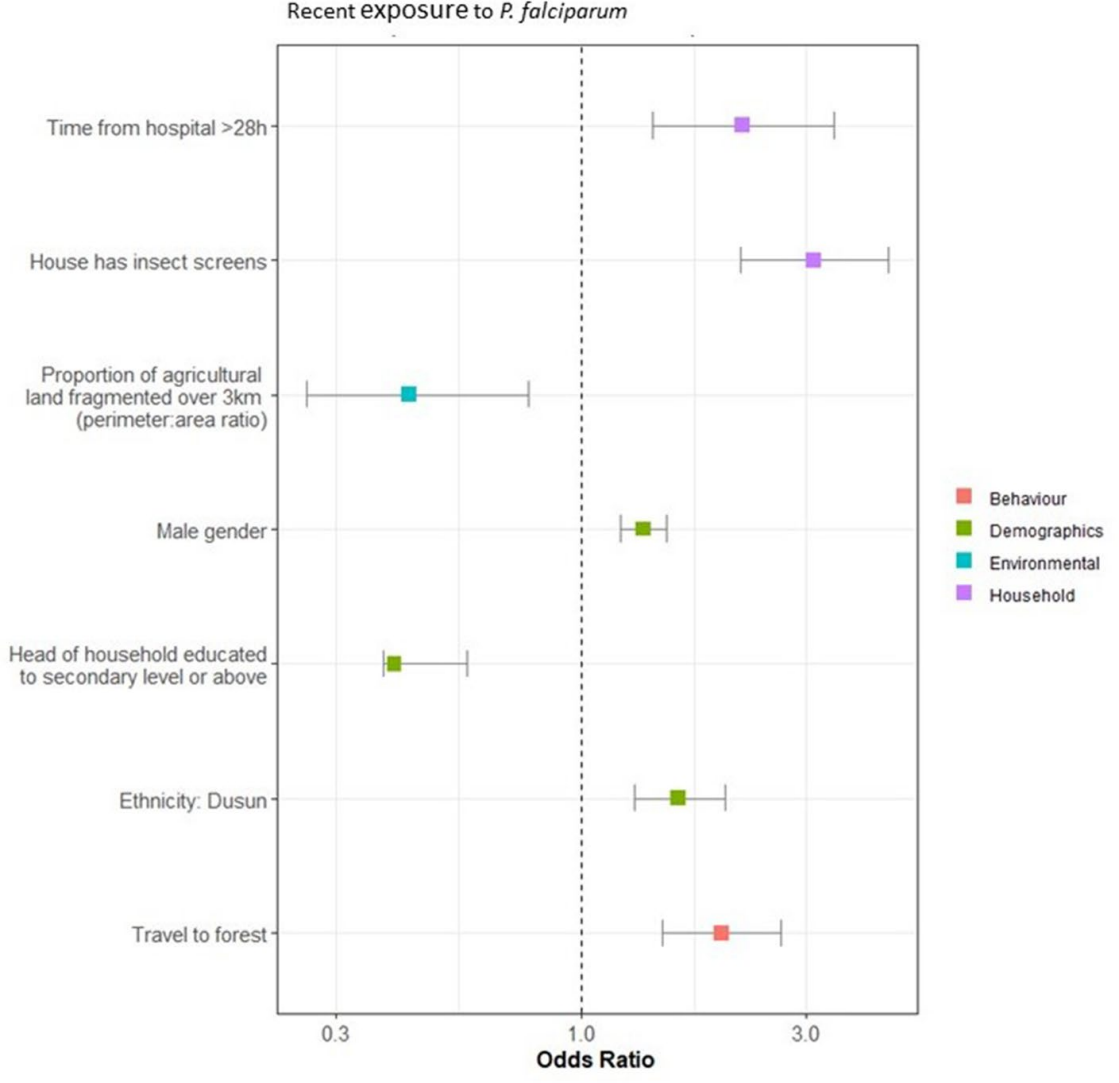
Results of the final multivariate model for risk factors for recent exposure to *P. falciparum*. Factors with an odds ratio > 1 are classed as risk factors (found to increase an individual’s likelihood of exposure), and factors with an odds ratio < 1 are classed as protective factors (found to reduce an individual’s likelihood of exposure).

**Figure 5:**
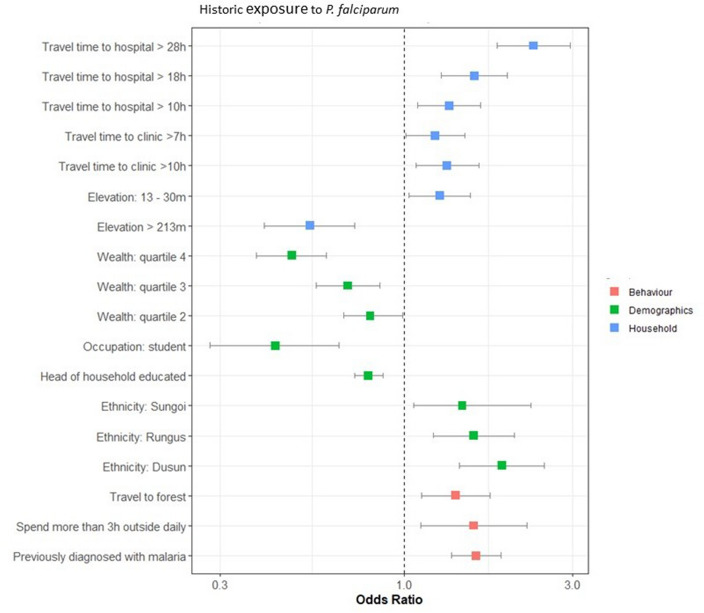
Results of the final multivariate model for risk factors for historic exposure to *P. falciparum*. Factors with an odds ratio > 1 are classed as risk factors (found to increase an individual’s likelihood of exposure), and factors with an odds ratio < 1 are classed as protective factors (found to reduce an individual’s likelihood of exposure).

**Figure 6:**
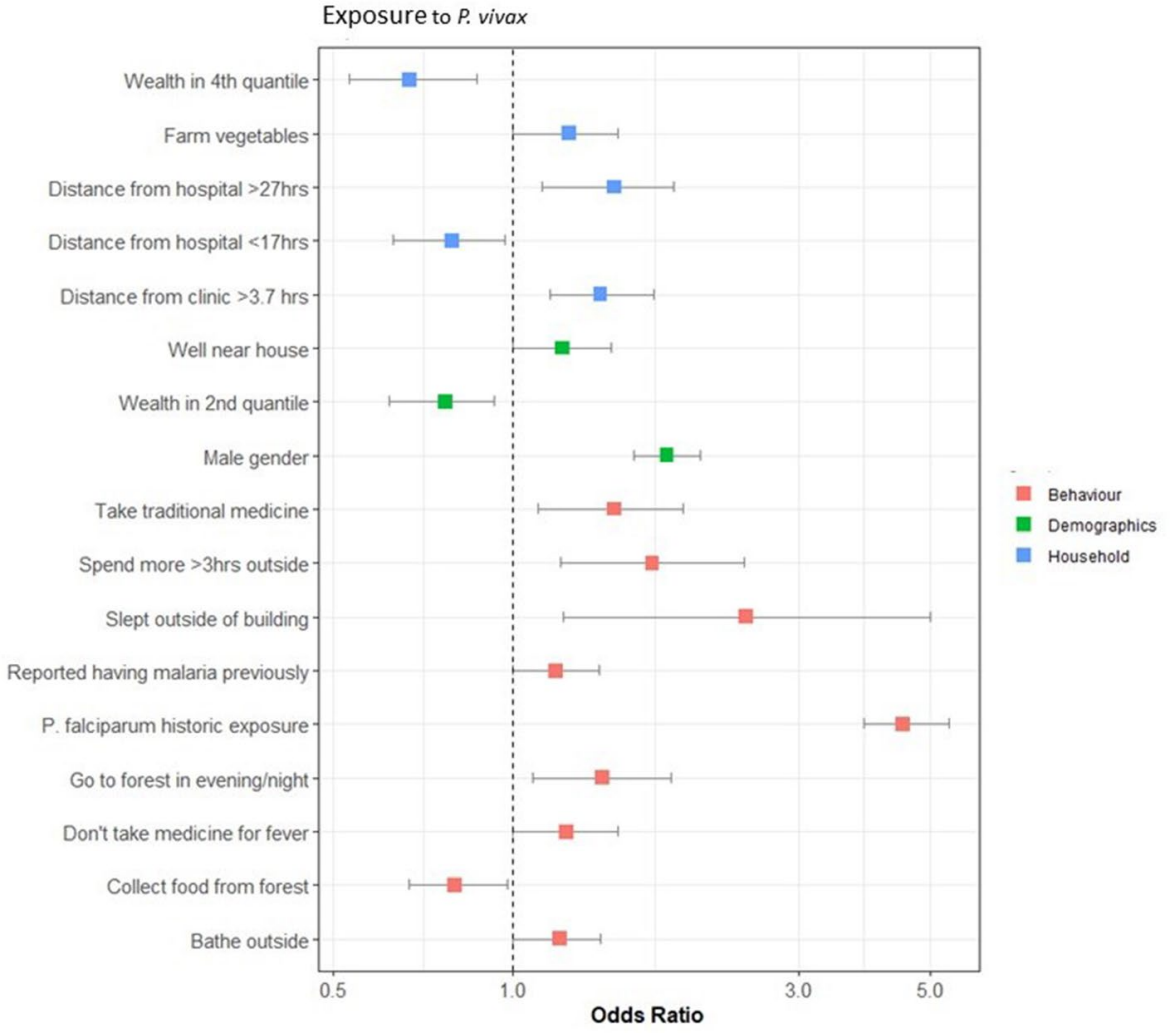
Results of the final multivariate model for risk factors for exposure to *P. vivax*. Factors with an odds ratio > 1 are classed as risk factors (found to increase an individual’s likelihood of exposure), and factors with an odds ratio < 1 are classed as protective factors (found to reduce an individual’s likelihood of exposure).

The results of the risk factor assessments for historic and recent exposure to *P. falciparum* and historic exposure to *P. vivax* are provided in Figs. 4, 5 and 6.

Higher travel times to hospitals and clinics were found to be a risk factor in all three assessments. Various forest activities were also found to be risk factors for both species and all exposures. Falling within higher wealth quantiles was a protective factor for historic exposure to both *P. falciparum* and *P. vivax,* and the head of the household being educated to a secondary level or above was a protective factor for recent and historic exposure to *P. falciparum*. Male gender was also a risk factor for exposure to *P. vivax* and recent exposure to *P. falciparum*.

### Environmental risk factors and spatial distribution of exposure

**Figure 7:**
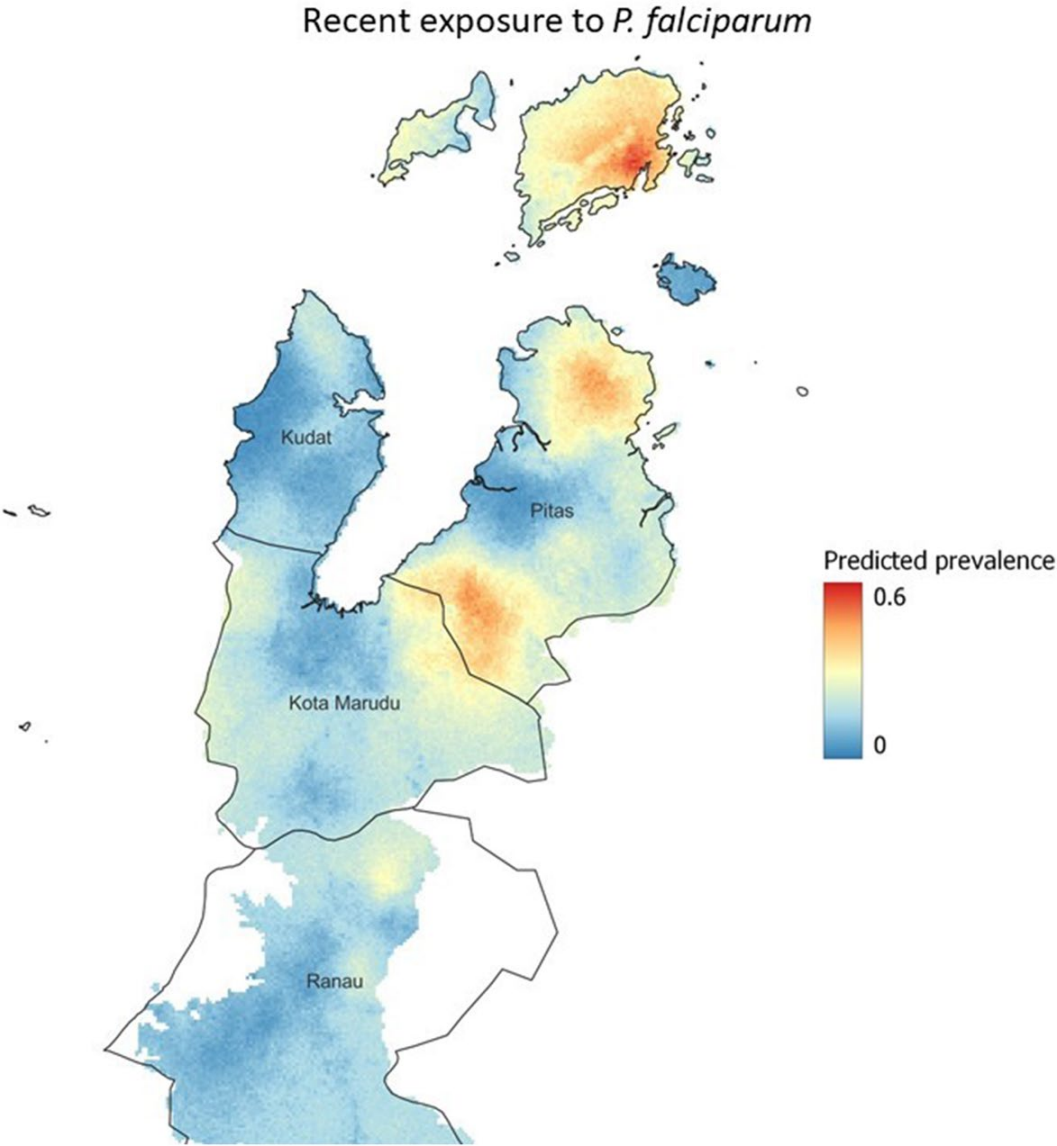
Geostatistical map showing mean posterior estimated prevalence of recent exposure to *P. falciparum* from serological markers. Maps were developed using R-INLA^25^ and visualised using QGIS (version 3.16.11)^24^.

**Figure 8:**
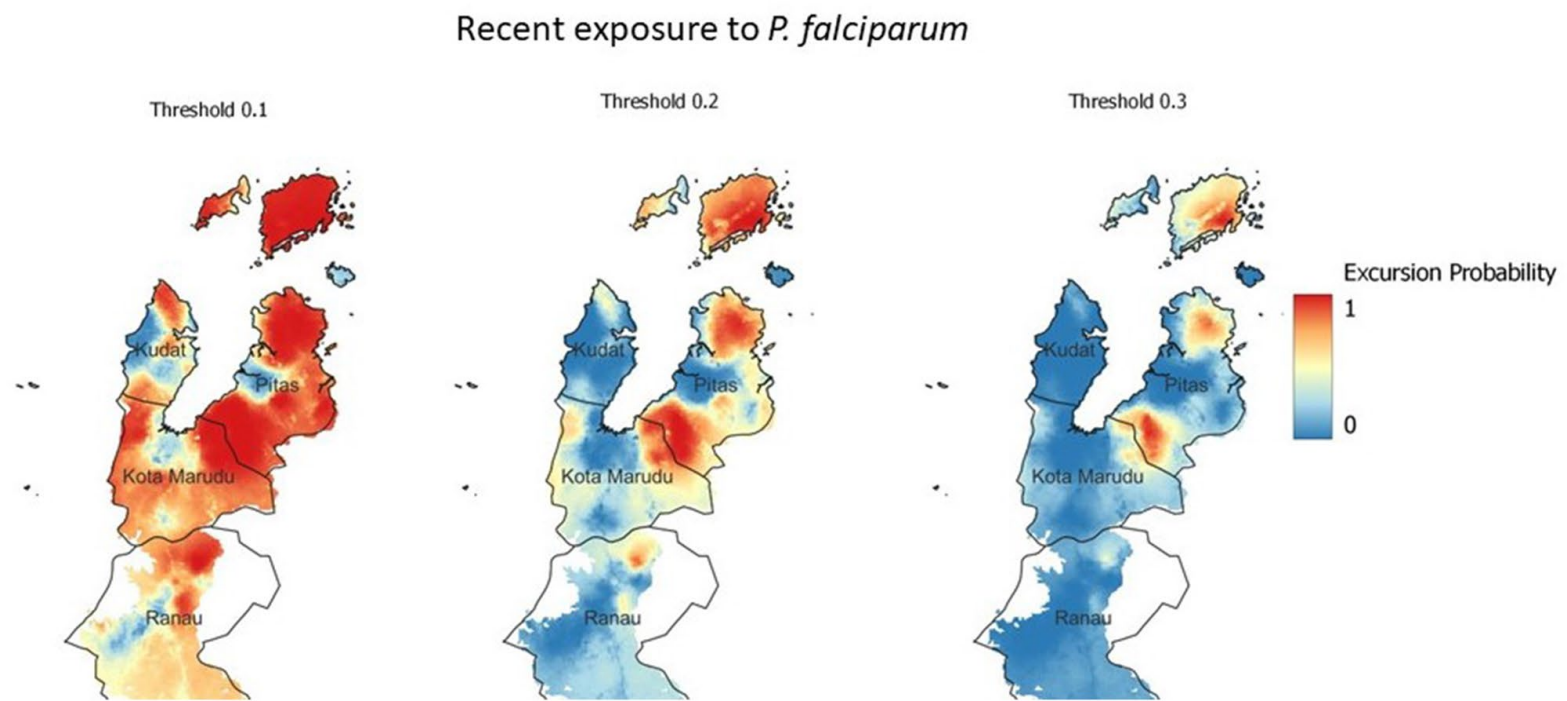
Exceedance probability using a 10% threshold, 30% threshold and 50% threshold (i.e. In the left hand image the probability that seroprevalence exceeds 10%). Maps were developed using R-INLA^25^ and visualised using QGIS (version 3.16.11)^24^.

We identified multiple bioclimatic variables, normalised vegetation index (NDVI), distance from old forest, agriculture and the sea, as environmental and spatial risk factors for household seroprevalence of recent exposure to *P. falciparum.* The inclusion of the spatial term was found to improve the fit of the geostatistical model (Supplementary Table 5). Figure 7 shows the map of predicted prevalence for recent exposure to *P. falciparum.* In addition to predicting the prevalence of exposure across the study area, the exceedance probabilities for each location were calculated. To investigate the impact of setting different prevalence thresholds, we produced exceedance probability maps at 10%, 20% and 30% seroprevalence (Fig. 8).

The PCR positive samples were too scarce to model geostatistically as a single outcome. Consequently, we produced predicted prevalence and exceedance probability maps for the combined outcome of current and recent exposure to *P. falciparum* from PCR and serology diagnostic endpoints (Supplementary Figs. 2 and 3).

## Discussion

The key objective of this study was to describe population-level exposure to the human malarias *P. falciparum* and *P. vivax* in Sabah, Malaysia at a time when the country was nearing elimination. Our predictions showed recent exposure to *P. falciparum* within the population to be very low, with exposure increasing with age. We found that forest activity and longer travel time to health facilities were common risk factors for recent and historic exposure to *P. falciparum* and *P. vivax.* Additionally, we produced prediction maps of recent exposure to *P. falciparum* within the study area in Sabah, and corresponding exceedance probability maps which showed 3 clear foci of exposure in the Pitas and Kota Maradu districts. This study demonstrates the application of MBA serology, machine learning, geostatistical methods and remote sensing derived data to characterise the exposure, risk factors and spatial distribution of human malarias in a pre-elimination setting.

Overall, the exposure predictions for historic and recent *P. falciparum,* and *P. vivax* align with knowledge of transmission in South–East Asia and specifically in Malaysia at the time of the study. Recent exposure to *P. falciparum* was very low in comparison to historic exposure and *P. vivax* exposure, which can be expected for a country in the pre-elimination stage^26^. The age-stratified seroprevalence for recent *P. falciparum* exposure was close to 0% for the survey population until 30 years of age, and not increasing past 15% in older populations. The higher levels of recent exposure in older age groups may be due to activities such as travelling, hunting and working in forested areas, which bring individuals into proximity with forest-dwelling malaria vectors. Such activities are typically performed by adults, resulting in higher exposure in older age-groups^27,28^. *P. vivax* exposure was higher than recent *P. falciparum* exposure. This is common as countries in Asia and the Americas near elimination, where *P. vivax* often contributes to more of the malaria burden than *P. falciparum.* Here, primary infections from mosquitoes drop and more cases are caused by reactivation of hypnozoite stage parasites^29^.

We produced geostatistical maps for recent exposure to *P. falciparum.* The results show recent exposure to be highly spatially heterogeneous, as is common in low-transmission and pre-elimination settings. We predicted three foci of high transmission, where there is a high probability that seroprevalence exceeds 30%. These exceedance probabilities are useful as they contextualise the predicted prevalence maps into units which are more interpretable for health-decision making^18^^,^^19^. These probabilities could be used by National Control Programmes to inform prioritisation of control or surveillance efforts. In this study we showed exceedance probabilities for seroprevalences of 10% 20% and 30%, however these thresholds could be set by control programmes according to their own criteria. One of the unique facets of serology is that it captures exposure over longer periods of time than infection metrics such as PCR and RDT^30^. We fit models for recent exposure using training data from individuals with known *P. falciparum* infections within the past two years, including recently infected individuals (e.g. exposed within the past week or month). Due to this, identified foci likely correspond to transmission within this time window. Longitudinal studies would be required to identify how foci change in the future, particularly with on-going malaria control activities.

The village-level clustering of PCR confirmed cases of *P. falciparum* and *P. vivax* suggests some focal outbreaks at the time of the survey. Genotyping of these samples may be interesting to investigate the inter and intra-village relatedness of parasites. The low numbers of PCR confirmed cases of *P. falciparum* and *P. vivax* limited our ability to use them in a joint model to investigate any shared spatial effect and common underlying processes between recent exposure and infection. We did, however, combine the exposure and infection results and produced a prediction map of ‘recent and current infection’. Overall, these maps predicted the same three hotspots of transmission, and an additional area of transmission in the south-east of the study area. There were no PCR confirmed cases in this additional area, and we suspect that these predictions were based off new associations between PCR results and environmental variables.

The age-stratified seroprevalence curves for all exposures follow the same trend of low exposure until around 20 years of age, with exposure increasing after this point. Typically, in South–East Asia, malaria is an occupational hazard for forest workers and exposure increases at around 20 years of age when males begin forest work. The risk of human (and zoonotic) malarias from forest activity in South-East Asia is well-documented^4,22,31–36^. We found that forest activities (travelling to forest and going to the forest in the evening) were common risk factors across exposures to recent and historic *P. falciparum,* and *P. vivax.* In Sabah, this is driven by the ecology of the shared primary vector *Anopheles balabacensis. An. balabacensis* is primarily reported as a forest-dwelling species^37^, although recent research has highlighted that the vector may be adapting to changing land-use patterns^38^. Collecting food from the forest was found to be a protective factor for exposure to *P. vivax*. This is interesting as it opposes the main findings from this paper and widely reported findings in the literature of forest and forest activities being risk factors for exposure. One explanation may be that individuals gather food during daytime when malaria vectors are biting less, resulting in less time spent in the forest during the evening biting hours of *An. balabacensis.* Additionally, primary and intact forests have lower vector densities^39^, and have also been found to be a protective factor for *P. knowlesi* in this area, so this finding may reflect differences in land type as well as behaviour. It is important to also acknowledge the low levels of human malaria transmission seen in this study, meaning the majority of *P. vivax* antibody responses are likely to be relapse cases, which can confound exposure patterns.

The second risk factor which was common between all exposures was longer travel times to health facilities (hospitals and clinics). Geographic distance or travel time to health facilities is often a significant predictor when modelling malaria prevalence and a significant factor for controlling burden^40–45^. This highlights the vulnerability of communities living in remote areas who are less able to seek access to healthcare when it is needed^46^. Improving equity in access to prompt diagnosis and treatment is vital to achieve and sustain elimination of malaria in these hard-to-reach communities^6^. We found other risk and protective factors which were common across more than one exposure type. Male gender was found to be a risk-factor for *P. vivax* and recent exposure to *P. falciparum*. Similarly, men were found to be at higher risk of exposure to human and zoonotic malarias in a previous case–control study in the area^47^. This suggests that the activities which bring males into contact with vectors are similar between zoonotic and non-zoonotic malaria, and the vectors for zoonotic and non-zoonotic malaria are the same or may occupy similar habitats. A higher level of education was found to be a protective factor against recent and historic exposure to *P. falciparum,* and wealth was found to be a protective factor in historic exposure to *P. falciparum* and *P. vivax.* Education and wealth are likely to be lower in the hard-to-reach communities described above. Forest-fringe environments with high malaria transmission are often inhabited by ethnic minority groups, migrant workers and workers involved in illegal or semi-illegal activities (logging, mining), experiencing a high degree of poverty and with difficulty accessing healthcare^6,48,49^. Promising solutions to the challenges of access to healthcare and health education may be mobile malaria clinics and village volunteer malaria workers^6^.

Environmental variables around the household were only included in the risk-factor analysis for recent exposure to *P. falciparum.* This is as it is unlikely that the environmental conditions at the time of the survey would affect an individual’s lifetime exposure. Individuals may have moved away from the location, and thus the environmental conditions, where they were historically exposed. We found that fragmentation of agricultural land over 3 km was a risk-factor for recent exposure to *P. falciparum.* Mixed agriculture was also found to be a predictive factor for the geostatistical mapping of recent *P. falciparum* exposure*,* highlighting how risk-factors around individual’s households translate to broader-scale patterns in transmission. Land-use change and fragmentation, and agricultural activities have been shown to be significant risk factors for zoonotic *P. knowlesi* in the study area^22^, which is transmitted by the same vector *An. balabacensis.* Habitat fragmentation and agriculture have also been shown to be associated with this vector’s breeding^50^.

There were some limitations in this study. At the time of classification of *P. vivax* exposure by Fornace et al*.*^22^, there was not sufficient machine-learning training data to differentiate between recent and historical *P. vivax* exposure. For this study we have therefore treated the *P. vivax* classification as a “general exposure” to *P. vivax.* More discriminatory antigens for defining time-specific exposures to *P. vivax* have since been developed^12^. The complex biology and epidemiology of *P. vivax* is the reason we did not perform geostatistical mapping on this exposure metric, as we were not capable of confidently defining recent exposure. Additionally, there were limitations to the risk-factor analysis. Our definition of historic exposure to *P. falciparum* is infection within the past 20 years, based on an estimated antibody half-life of 20 years. However, participants’ answers likely reflect their behavior at and around the time of the survey in 2015. Therefore, the ‘risky’ behaviors they report in the questionnaire may not be those which resulted in their historical exposures. Similarly, behaviors reported in the survey may not have resulted in primary *P. vivax* exposures, and serological signals from relapses may influence these results. These limitations should be considered when interpreting the risk factors for historic exposures to *P. falciparum* and exposure to *P. vivax*. There were also some limitations to the training data used to classify sero-negative individuals. Antigenic variation and variation in the human immune system may limit how well the negative reference population of naïve individuals from a non-endemic area compare with exposed individuals from the study area^51^. While it would have been ideal to use training data of known exposure status from the geographic region of the study site, the continued transmission of *P. falciparum* and *P. vivax* made it impossible to identify unexposed individuals within the study site.

Despite some limitations, there is huge potential for the use of these methods to supplement surveillance activities in elimination settings. Serological surveillance is increasingly being used to monitor elimination efforts in low transmission settings where the probability of detecting active infections is low. Integrating this into geostatistical frameworks enables identification of areas with the highest probability of recent transmission and prediction across wider geographical areas. Most of the Earth Observation-derived data is free, frequently available and easily accessible, therefore the analysis could be repeated if serological data were available. MBA serological surveys are relatively low-cost and logistically feasible to supplement existing surveillance activities. Additional approaches have explored using serological surveillance with convenience sampling methods to collect community level data at relatively low cost^52^. A key characteristic of MBA is the ability to assess exposure to multiple pathogens simultaneously. This study and supporting studies from the same serological survey on Neglected Tropical Diseases (NTDs) by Chan et al.^53^*,* and zoonotic malaria by Fornace et al.^22^ demonstrate the wealth of information which can be yielded from integrating serology with geostatistical methods, and how useful the outputs of such studies can be.

Although this is a retrospective analysis of data which was collected 7 years prior to the time of writing, in a country which has now effectively eliminated local transmission of *P. falciparum* and *P. vivax,* the findings are still useful. This study sheds important insights into the individual and household-level risk factors for human malaria exposures in Sabah during the pre-elimination phase. We showed that forest activities and distance from health facilities were significant risk factors for exposure across *P. falciparum* and *P. vivax*. We also produced predictive maps of seroprevalence for recent exposure to *P. falciparum,* based on environmental predictors. Additionally, we built on previous work by Fornace et al*.*^22^ using machine learning methods to predict exposures from MBA serology data. The ability to estimate recent exposure to *P. falciparum* allowed for environmental and spatial risk factors for ongoing transmission to be assessed, and for meaningful geostatistical modelling to be performed. Our results may be useful to inform policy makers and surveillance teams of common risk factors for human malaria, and areas which may be receptive to outbreaks in the unfortunate event of a resurgence of transmission.

## Methods

### Study site

The study took place from September 17th to December 12th 2015 across the Kudat, Ranau, Pitas and Kota Marudu districts of Northern Sabah, Malaysian Borneo. The climate in the region is tropical and elevations range from sea-level to over 4000 m above sea-level. The combined population of these districts was 280,000 at the time of the study, situated predominantly in rural locations and with most occupations being associated with agricultural or plantation activities.

### Study design and sample collection

An environmentally stratified, population-based cross-sectional survey was conducted, as described by Fornace et al. ^22^. A non-self-weighting two-stage sampling design of 919 villages was used to estimate seroprevalence to various malaria species; this analysis focuses on antibody responses to antigens from the human malaria species *P. falciparum* and *P. vivax.* The villages were stratified by forest cover, with a target sample size of 2650 households. All individuals who had been residing in selected households for the past month were asked to participate. Finger prick blood sampling was performed to prepare blood smears for the detection of malaria parasites by microscopy, and whole blood spots were collected using finger-prick blood sampling and EDTA microtainers (BD). Samples were spun to separate plasma and red blood cells and stored at − 20 °C. Participants also completed a questionnaire survey on individual and household information including demographic, health and socio-economic indicators. Location data was recorded for all households using a handheld GPS receiver (Garmin, USA).

### Laboratory procedures

Laboratory procedures for the analysis of samples collected during the study are described by Fornace et al.^22^. For the molecular identification of infection, DNA was extracted from whole blood samples. Samples were pooled into 10 × 10 matrices with 40 μl of each sample loaded on one vertical and one horizontal pool. QIAsymphony DNA Midi kit (Quiagen, UK) were used to extract the 400 μl pools on a QIAsymphony SP/AS instrument (Quiagen, UK). The pools were eluted in 200 μl of elution buffer provided within the kit. The extracted DNA pools were amplified by genus-specific 18S-ribosomal DNA nested PCR as described by Singh^54^. The nested PCR products were analysed on 1.5% agarose gels, and genus-positive sample pools were de-pooled and reamplified. Positive samples were visualised on agarose gels and speciated using methods described by ^54,55^.

Immunoglobulin G responses to 16 non-zoonotic malaria parasite antigens were measured: *Plasmodium falciparum* glutamate-rich protein (GLURP-R2), early transcribed membrane protein (Etramp) 5, gametocyte exported protein (GEXP18), merozoite surface protein (MSP)2-Ch150/9, MSP2-Dd2, apical membrane antigen 1 (AMA1), MSP1–19, and schizont egress antigen (SEA)-1; *Plasmodium vivax* MSP-1, erythrocyte binding protein (EBP), Duffy binding protein (DBP) RII, and DBPII.* P knowlesi* and *P. vivax* AMA-1 were excluded from the analysis due to possible cross-reactivity with *Plasmodium vivax* antigens^56^. Luminex (Luminex, Austin, TX, USA) magnetic microsphere conjugation was done by standard methods. Results were read using the LuminexMaGPIX system, measuring quantitative antibody responses measured as median fluorescent intensity (MFI). Sera from confirmed clinical cases of *P. falciparum* and *P. vivax* as described by Herman et al*.*^56^ were used as positive control pools, and serial dilutions of the positive control pools for were used to generate standard control curves.

### Classification of exposure

All analysis was performed in R statistical software^57^.

To classify individuals as seropositive for recent exposure to *P. falciparum*, we used an ensemble approach for binary classification with the Super Learner algorithm^58^. This algorithm estimates the performance of multiple machine learning models by cross validation, then creates an “ensemble” optimal weighted average of those models ^59^. The machine learning models included in the ensemble were support vector machine (SVM), random forest (RF), lasso, K-nearest neighbour (KNN) and boosted regression tree (BRT). Weights for base learners were calculated using the Nelder-Mead method to maximise the Area Under the ROC Curve (AUC).

The algorithms were trained with datasets of antibody responses from individuals with a known exposure to, or concurrent infection with *P. falciparum*, and negative controls from immunologically naïve individuals from the UK and USA (Supplementary Information Table 1)*.* To avoid overfitting, we used a random 70% of the dataset to build the model, leaving the remaining data for independent validation. The full dataset with tenfold cross-validation was used to make predictions. The outcome was a prediction of probability of recent exposure to *P. falciparum*. Individuals with a probability of > 0.5 were classed as positive to infection within the last 2 years, as training data included cases within this timeframe. This is hereby referred to as “recent exposure”.

To specify the combination of antigens included in the final classification model, the relative influence of each antigen in the prediction of positivity for recent exposure to *P. falciparum* was calculated using a BRT algorithm. The SuperLearner ensemble was run on combinations of 3 to 8 antigens, beginning by dropping the antigen of least influence. The final combination was taken as the most parsimonious model with the highest predictive power, i.e., the lowest number of antigens which yielded the highest AUC.

Historic exposure to *P. falciparum* and exposure to *P. vivax* were classified as described by Fornace et al*.*^22^*,* using the SuperLearner methodology with different training data and combinations of antigenic targets*.* To classify historic exposure to *P. falciparum* they used molecularly confirmed cases from Northern Sabah taken up to a year after diagnosis^47^, and longitudinal samples from individuals over 5 years old in a previously hyper-endemic area which was experiencing massive reductions in tramsission^60^. To classify exposure to *P. vivax*, training data for sero-positives included molecularly confirmed *P. vivax* infections^47^, confirmed *P. vivax* exposed individuals from endemic areas in Ethiopia and Brazil^56^, and positive *P. vivax* controls. Negative reference populations for both species consisted of samples from UK residents with no history of travel.

### Risk factor assessment

Individual-level demographic, health and socio-economic risk factors for recent exposure to *P. falciparum*, historic exposure to *P. falciparum*, and historic exposure to *P. vivax* were assessed. Additionally, remote-sensing derived environmental risk factors for recent exposure to *P. falciparum* over multiple spatial scales (100 m–5 km) were also investigated. All risk factors investigated are detailed in Fornace et al.^22^. Age-adjusted univariate binomial mixed-effect logistic regression models were run for each covariate against each exposure classification, with household included as a random effect to account for spatial autocorrelation. Variables with *p* < 0.2 were included in the multivariate analysis. The final age-adjusted multivariate model was developed using a forward-stepwise logistic regression procedure, where all variables significant at *p* < 0.05 were retained, and each step was assessed to ensure reduction in Akaike Information Criterion (AIC). Factors with an odds ratio > 1 were classed as risk factors (found to increase an individual’s likelihood of exposure), and factors with an odds ratio < 1 were classed as protective factors (found to reduce an individual’s likelihood of exposure).

### Mapping exposure risks

The spatial distribution of the household prevalence of recent exposure to *P. falciparum* was assessed and a prediction map was developed using Earth Observation-derived data. Potential environmental and spatial covariates including climatic variables, accessibility to hospitals and clinics, population density, distance to various types of land cover and forest, and topographic measures, were assembled (Supplementary Information Table 4.) Demographic data was not available across all locations within this region, therefore questionnaire survey data was not included.

Geostatistical models of household prevalence were fit in a Bayesian framework where ${p(x}_{i})$ denoted the seroprevalence at locations ${x}_{i}, i=1\dots n$, with ${m}_{i}$ individuals sampled per household location. The full model was specified as:

$${Y}_{i} \sim \mathrm{Binomial}\left({m}_{i}, {p(x}_{i})\right)$$

With the linear predictor for the binomial model specified as:

$$\mathrm{logit}\left({p(x}_{i})\right)= {\beta }_{0}+{{\varvec{d}}({\varvec{x}}}_{{\varvec{i}}})\boldsymbol{^{\prime}}{\varvec{\beta}}+ {w}_{i}$$

where ${\beta }_{0}$ represents the intercept, ${\beta }_{0}+{{\varvec{d}}({\varvec{x}}}_{{\varvec{i}}})\boldsymbol{^{\prime}}{\varvec{\beta}}+ {w}_{i}$ represents a vector of location-specific covariate effects, and ${w}_{i}$ represents the spatial effect. The stochastic partial differential equation (SPDE) approach was used to model the spatial effect as a Matern covariance function. The model was built and implemented in Integrated Nested Laplace Approximation (R-INLA)^25^. Penalised complexity priors were used for the spatial effect and weakly informative priors were used for the fixed effect coefficients and intercepts^61^. Deviance Information Criteria (DIC) was used to assess final model fit. The posterior probabilities for final model predictions were estimated from 1000 posterior samples. The uncertainty around predictions were visualised as exceedance probabilities for seroprevalence at a 10%, 20% and 30% threshold. These maps represent the probability that a location exceeds each seroprevalence threshold, where areas with exceedance probability around 50% are highly uncertain, above 50% are increasingly likely to be above the threshold, and below 50% are decreasingly likely to be positive.

Although village-levels clusters of positive results were observed, the number of positive pooled PCR results were too sparse for geostatistical modelling. To investigate the spatial distribution of a combined outcome for current infection and recent *P. falciparum* exposure, we combined the PCR and serology results and ran geostatistical models as described above.

### Ethics

This study was approved by the Medical Research Sub-Committee of the Malaysian Ministry of Health (NMRR-14-713-21,117) and the Research Ethics Committee of the London School of Hygiene and Tropical Medicine (8340). Written informed consent was obtained from all study participants, and all methods were performed in accordance with the relevant guidelines and regulations.

### Supplementary Information

Supplementary Information.

## Data Availability

The datasets used and/or analysed during the current study available from the corresponding author on reasonable request.
