# Supplementary material for: Serological evaluation of risk factors for exposure to malaria in a pre-elimination setting in Malaysian Borneo
Source: Sci Rep. 2023 Aug 10;13:12998. doi: 10.1038/s41598-023-39670-w (PMC10415323; doi:10.1038/s41598-023-39670-w)
Supplement: Supplementary file 1 — Supplementary Information. [file 41598_2023_39670_MOESM1_ESM.docx]

Supplementary Information

Supplementary Information Table 1: Training and validation data used in models for classification of recent exposure to P. falciparum

| Location | Diagnostic type | *P. falciparum* positive | *P. falciparum* negative | Total samples |
| --- | --- | --- | --- | --- |
| Malaysia | PCR | 47 | 5 | 52 |
| Philippines | Standard controls | 4 | 0 | 4 |
| Philippines | PCR/RDT/Microscopy | 568 | 0 | 568 |
| Philippines | < 50 years old in no-transmission setting | 0 | 550 | 550 |
| Malaysia | PCR | 17 | 0 | 17 |
| United Kingdom | Immunologically naïve | 0 | 18 | 18 |
| United Kingdom and USA | Immunologically naïve | 0 | 510 | 510 |
| Total | | 636 | 1083 | 1719 |

Supplementary Information Table 2: Relative influence of P. falciparum antigens on the prediction of positivity for recent exposure

| Antigen | Relative Influence |
| --- | --- |
| GLURP.R2 | 64.12169 |
| AMA1 | 18.24557 |
| MSP1-19 | 8.182692 |
| Etramp5 | 6.907912 |
| GEXP18 | 1.581911 |
| SEA | 0.631227 |
| MSP2-Ch150/9 | 0.176893 |
| MSP2-Dd2 | 0.15211 |

Supplementary Information Table 3: Results of ensemble and individual algorithms on the prediction of recent exposure to P. falciparum

| Algorithm | AUC | Min | Max |
| --- | --- | --- | --- |
| Super Learner | 0.99658 | 0.974138 | 1 |
| RF | 0.986207 | 0.862069 | 1 |
| BRT | 0.997874 | 0.987069 | 1 |
| SVM | 0.979409 | 0.828571 | 1 |
| KNN | 0.964152 | 0.87069 | 1 |
| Lasso | 0.977586 | 0.775862 | 1 |

Supplementary Information Table 4: Spatial and environmental covariates

| Parameter | Description | Resolution | Source |
| --- | --- | --- | --- |
| Elevation | Elevation (metres above sea level) | 30m | ASTER Global Digital Elevation Map^54^ |
| Slope and aspect | Slope and aspect (degrees) | 30m | Calculated from ASTER Global Digital Elevation Map |
| TWI | Topographic wetness index | 30m | Calculated from ASTER Global Digital Elevation Map |
| NDVI | Normalised differential vegetation index | 30m | Calculated from NIR and Red Landsat8 bands^55^ |
| Land cover | Euclidean distance to different land cover types | 5m | Calculated from land cover map of study site, described by^21^ |
| Location of houses | GPS coordinates | 30m | Calculated from land cover map of study site, described by^21^ |
| Distance to roads and houses | Distance from nearest road and house | 5m | Calculated from GPS coordinates |
| Population density | UN-adjusted 2015 population density | 30m | World Pop^56^ |
| Bioclimatic variables | Bioclimatic indicators of ecology, 1970 - 2000 | 30m | Calculated from^57^ |


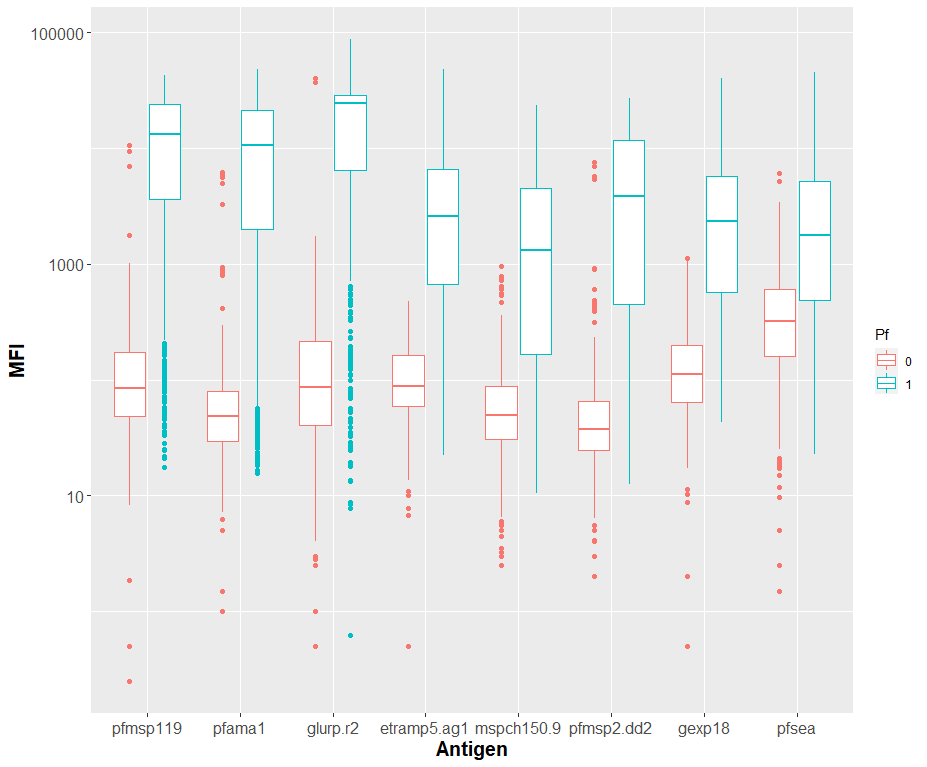


Supplementary Figure 1: MFI antibody responses (log10 scale) of training data used for classification in known positive (1) and negative (0) individuals for recent exposure for P. falciparum. The line in the middle of each box represents the median value and the lower and upper hinges correspond to the first and third quartiles (the 25th and 75th percentiles).

Supplementary Information Table 5: Mean posterior estimates of coefficients of fixed effects, including 95% Bayesian credible intervals

|  | **95% BCI** | | |
| --- | --- | --- | --- |
| **Covariate*** | **Mean** | **2.5%** | **97.2%** |
| NDVI | 0.132 | 0.021 | 0.243 |
| Distance from mixed agriculture | -1.549 | -2.881 | -0.217 |
| Distance from intact forest (based off canopy cover threshold ^21^) | -0.391 | -0.632 | -0.150 |
| Euclidean distance from the sea | 2.464 | 0.630 | 4.296 |
| Average temperature | -3.058 | -6.363 | 0.244 |
| Maximum temperature | 1.883 | -0.473 | 4.238 |
| Mean diurnal temperature range | -2.337 | -4.087 | -0.590 |

* All covariates mean-scaled and centered


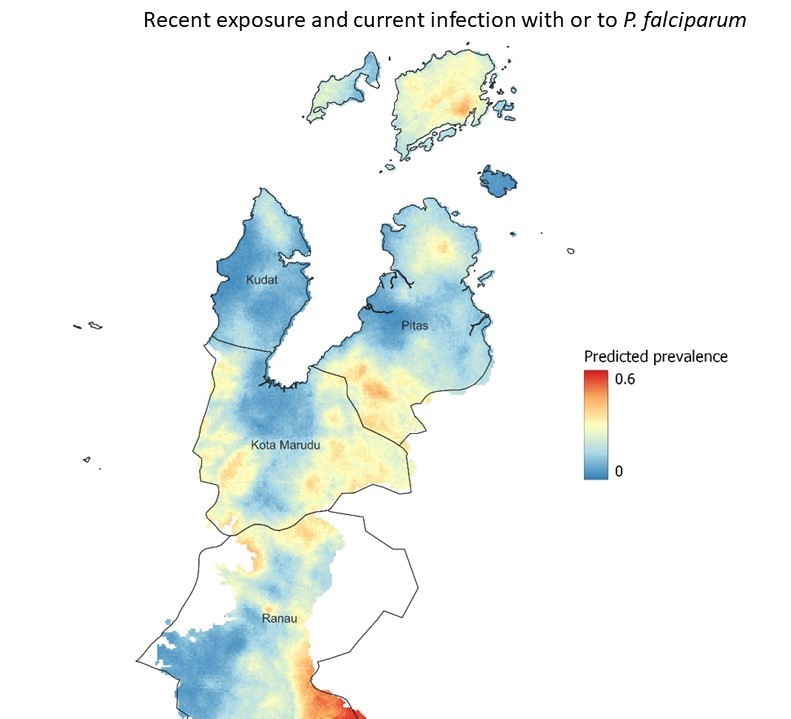


Supplementary Figure 2: Geostatistical map showing mean posterior estimated prevalence of recent exposure or current infection with or to P. falciparum from serological markers and PCR


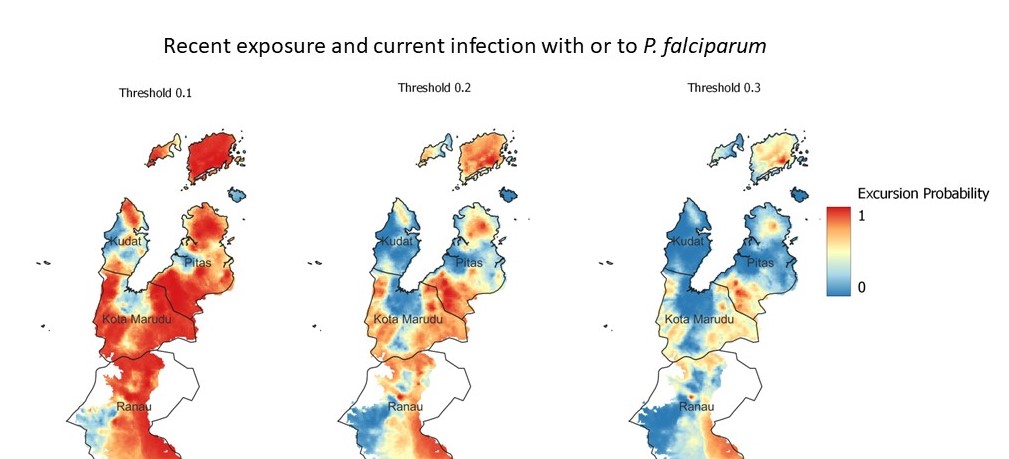


Supplementary Figure 3: exceedance probability for P. falciparum exposure or infection defined by serology and PCR diagnostics. Using a 10% threshold, 30% threshold and 50% threshold (i.e. the probability that exposure exceeds 10%)
